# Exfoliated Nanographite Inorganic-Based Composite Using Microfluidization

**DOI:** 10.1021/acsomega.5c08923

**Published:** 2025-10-27

**Authors:** Deborah M. Ciriaco, Paloma E. S. Pellegrini, Mara A. Canesqui, Silvia V. G. Nista, Stanislav Moshkalev

**Affiliations:** Center for Semiconductor Components and Nanotechnology, 28132 Universidade Estadual de Campinas, Campinas 13083-870, Brazil

## Abstract

Producing a few layers of nanographite flakes while maintaining micrometer-scale lateral dimensions remains a long-standing challenge. Conventional chemical and mechanical exfoliation methods are often costly, toxic, and difficult to scale, and they might also cause structural defects and debris. Our work addresses these issues, providing a scalable and cleaner route to high-quality exfoliated nanographite by using microfluidization. Through combined sequential processes of ultrasonication and microfluidization, we developed a methodology to produce exfoliated graphite flakes down to dozens of graphene layers while preserving the original micrometric sheet sizes. With the proposed methodology, it is also possible to control the concentration of the exfoliated nanographite obtained. Finally, we demonstrate the successful incorporation of the exfoliated nanographite into an inorganic-based composite based on sodium silicate, which ensures strong surface interaction and enables the fabrication of uniform thin films. The development of this composite expands the functional scope of exfoliated graphite for applications in coatings, optics, and photonics.

## Graphical abstract

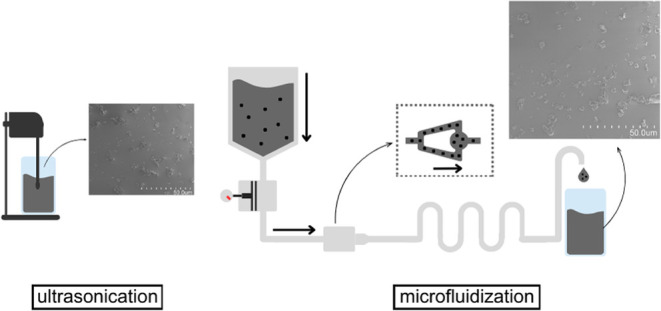

## Introduction

A promising approach to reduce the thickness of graphite flakes down to the nanoscale is the exfoliation of graphite.^1^ It is a green and scalable process^2^ that can be performed at room temperature.^3^ Exfoliated thin flakes preserve graphite’s desirable properties such as optical, thermal, and electrical.^4^ Moreover, the cost-effectiveness of exfoliation techniques enables applications in both academic research and industrial production.^3^

Significant effort has been devoted to developing efficient exfoliation techniques, in particular, top down approaches.^5^ Mechanical exfoliation techniques such as milling^6^ and roll-to-roll processing^7^ present high-throughput but face limitations in precise thickness control. In contrast, liquid-phase exfoliation (LPE) typically presents better thickness control. The LPE techniques rely on overcoming the van der Waals interaction between graphite layers by peeling micrometer-sized precursor graphite flakes in a liquid medium such as water or isopropyl alcohol. Among the most established LPE techniques, we can mention ultrasonication^8^ and high-pressure homogenization approaches such as microfluidization.^9–13^ Ultrasonication, in particular, can yield few-layer and even monolayer graphene. It is a process driven by cavitation, the formation, growth, and violent collapse of microbubbles. As cavitation is highly localized, it often results in small sheet sizes and large amounts of debris, leading to undesired excessive fragmentation.^12,14^

Microfluidization has recently emerged as a promising alternative within LPE techniques.^11–13,15^ In this process, a micrographite suspension is accelerated through an interaction chamber within a microchannel at pressures of up to 23000 psi.^16^ The suspension experiences intense shear forces, collisions, and, in some cases, localized cavitation, enabling efficient exfoliation. Microfluidization stands out for its scalability, low cost, and reduced number of defects. It is also a smoother exfoliation technique compared with cavitation-based processes. However, the use of exfoliated graphite via microfluidization in functional composites remains underexplored.

For a graphite composite to be functional, two key factors should be considered: the control over the concentration of exfoliated graphite in the composite and the interaction between the composite and the target substrate. Properties such as wettability, adhesion, and surface coverage play a critical role in using this type of composite for device fabrication.

Inspired by previous studies of functional graphite films on glass substrates,^17^ we propose, in this work, the exfoliation of graphite by microfluidization using deionized (DI) water and surfactant as the liquid medium. With this process, we successfully achieved exfoliated flakes with average thicknesses of 7 nm. Then, the exfoliated nanographite was combined with sodium silicate (Na_2_SiO_3_) to form an inorganic-based composite. Sodium silicate is a promising inorganic matrix for thin-film fabrication compared to conventional polymer-based matrices. Also known as aqueous vitreous, Na_2_SiO_3_ is semitransparent, compatible with flexible materials and it offers attractive combinations of properties for photonic applications.^18^ In addition, as sodium silicate promotes adhesion to substrates, we demonstrated how the exfoliated nanographite composite can be deposited to form stable films with nanometric thicknesses.

This article is organized into three sections. In the first section, we detail the preparation of the exfoliated graphite composite, including the precursor dispersion, microfluidization process, and incorporation of sodium silicate. In the Results and Discussion section, we report and discuss the flake morphology, surface interactions, and thickness reduction of exfoliated graphite. In the last section, we present our conclusions and perspectives.

## Experimental Section

The preparation of the exfoliated graphite composite was divided into three steps. Initially, we obtained the precursor graphite suspension. In sequence, this suspension was used to exfoliate graphite using microfluidization. Lastly, we present the exfoliated nanographite inorganic-based composite formulation by incorporating sodium silicate.

### Materials

We used micro graphite powder (Micrograf 99503 UJ, provided by Nacional de Grafite, Brazil) with a nominal sheet size of 3 μm and thickness of approximately 300 nm.^15^ For the liquid medium and composite preparation, we utilized surfactant Triton X-100 (Sigma-Aldrich, Germany), DI water, and sodium silicate (Sigma-Aldrich).

### Graphite Precursor Suspension

The standard preparation of precursor suspensions for exfoliation using the microfluidization technique typically employs isopropyl alcohol as a liquid medium.^13,19^ However, we found that sodium silicate is not compatible with isopropyl alcohol, as it forms spurious gel structures. Thus, we propose using DI water and a surfactant as the liquid medium for the graphite precursor suspension. After exfoliation of the precursor suspension, sodium silicate was incorporated to promote surface adherence and form the inorganic-based composite.

To begin with, we prepared a suspension with graphite powder in DI water at a concentration of 0.05 % in mass weight. The suspension was homogenized with an Ultra-Turrax disperser (T25, from IKA, Germany) for 30 min at 8000 rpm After homogenization, the suspension was treated with a probe ultrasonic processor (UP400S, Hielscher, Germany; 400 W, 24 kHz) to initiate the exfoliation process.

While previous studies have discussed the influence of sonication on exfoliation during microfluidization,^15,19^ here we propose applying probe ultrasonication prior to microfluidization. This pretreatment step partially exfoliates the flakes and only slightly reduces their lateral size and thickness. This reduction avoids occlusions in the microfluidization channels, which is a common issue with the method.^11^

For this investigation, probe ultrasonication was applied at 50% of the power for two duration processes: 5 and 30 min. The average lateral sheet size of 50 graphite flakes was measured for each duration of processing, and the results are exhibited in a Gaussian-like distribution in Figure 1. The vertical axis of the graph indicates flake counts, while the horizontal axis indicates the lateral sheet size of the flakes. Orange and green bars correspond to the sonication times of 5 and 30 min, respectively. The blue bars indicate raw flakes that were not subjected to sonication.

**Figure 1:**
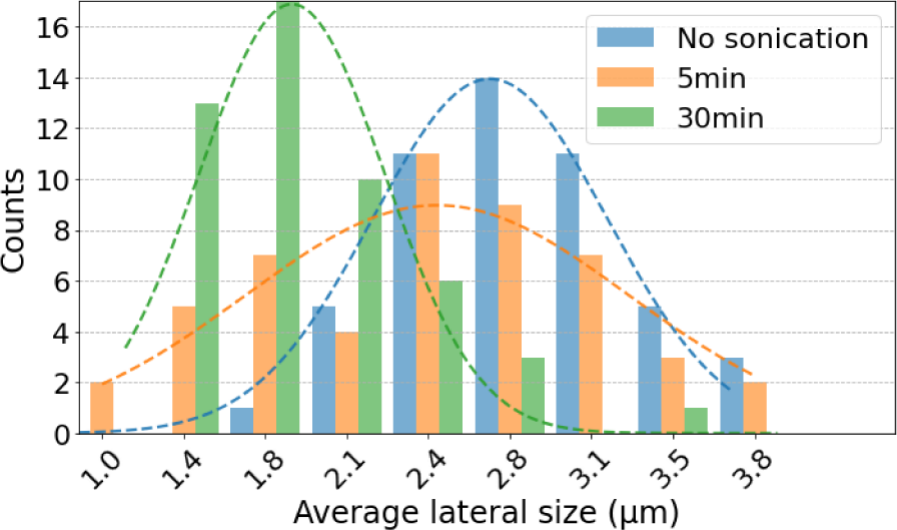
Gaussian-like frequency distributions of average sheet sizes of graphite flakes in suspension. The blue bars indicate the lateral size of raw graphite flakes, while the orange and green bars show the average size after probe ultrasonic processes for 5 and 30 min, respectively. A Gaussian-like fit was included to better notice the most frequent count of flakes for each processing.

From these measurements, we determined the mean sheet size and full width at half-maximum (fwhm) of the graphite flakes, as summarized in Table 1. Considering the flakes without sonication (blue bars), we can see a prominent bar at approximately 2.83 μm. The larger amount of flakes with this sheet size agrees with the nominal value of the purchased graphite powder (3 μm). After 5 min of sonication, we can see a broader distribution of the size of the flakes (orange bars). This broad distribution indicates that the sonication started to break the flakes, hence reducing their sheet size. When the flakes were subjected to 30 min of sonication, we observe a bar with greater height at 1.73 μm, indicating that sonication significantly reduced the sheet size compared to the nonsonicated flakes. Hence, the sonication process should be performed with care because it assists in exfoliation, but it also breaks the flakes and damages the surface. Such a reduction in sheet size impairs homogeneous thin-film formation, where flakes with large sheet size and low thickness are desired.

**Table 1:** Influence of Sonication on the Lateral Size of Graphite Flakes

Sonication time (min)	Size (μm)	fwhm (μm)
Nonsonicated	2.83	1.16
5	2.39	2.23
30	1.73	0.88

Our main objective with probe ultrasonication was specifically to reduce the flake thickness. A decrease in sheet size is undesirable, as it can limit potential applications. Moreover, we observed that graphite flakes with sheet sizes significantly smaller than 2 μm, similar to those obtained after 30 min of sonication, tended to cause more frequent capillary occlusions during the subsequent microfluidization. Considering these factors, we determined that a probe ultrasonication time of 5 min provides the optimal balance between initiating the exfoliation process and minimizing lateral size reduction.

To prevent agglomeration of graphite flakes, we introduced 0.2% of the surfactant Triton X-100 to the suspension. Then, the suspension containing graphite in DI water and surfactant was subjected to ultrasonic bath treatment for 3 min to promote dispersion of the nanographite. Following this step, the suspension was processed through microfluidization.

### Exfoliation by Microfluidization

The diagram in Figure 2 illustrates the methodology applied for the exfoliation of graphite flakes in suspension using the LM10 Microfluidizer Processor (Microfluidics, USA).

**Figure 2:**
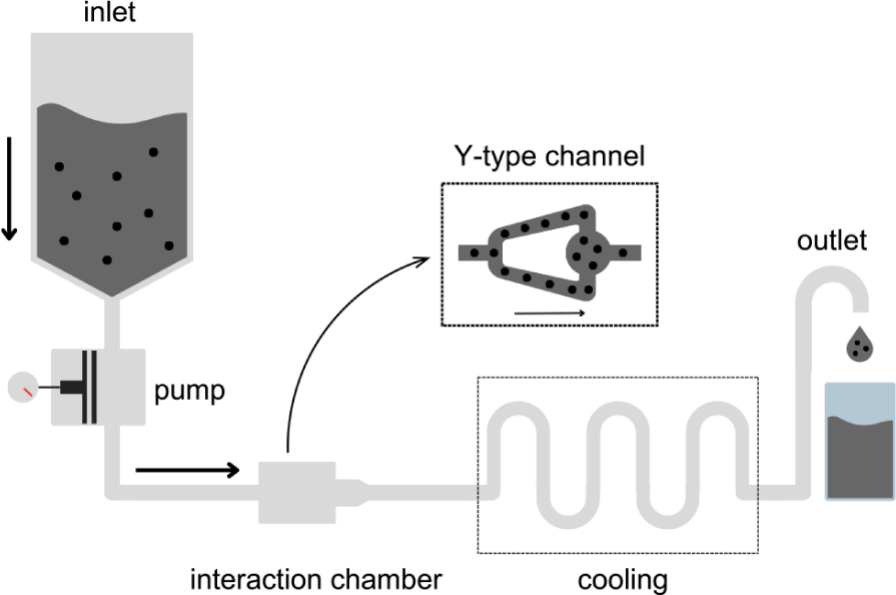
Microfluidization process: The precursor suspension is pressurized by an intensifier pneumatic pump and directed to the interaction chamber, which contains a Y-type channel responsible for the exfoliation process. Then, the exfoliated graphite suspension passes through a cooling serpentine before exiting the system. Original image is by the authors.

The precursor suspension, prepared as previously described, was loaded into the inlet. An intensifier pneumatic pump piston pressurized the suspension at 5000 psi and directed it into the interaction chamber in small volumes to prevent channel occlusions and ensure homogeneous treatment. By using 5000 psi instead of the higher standard pressures,^12,15^ we prevent excessive cavitation effects. The chamber features a Y-type geometry in which two opposing jets are generated as the fluid flows through parallel microchannels (75 μm in diameter). Inside the microchannels, the pressurized mixture of graphite, water, and surfactant undergoes high shear forces, interparticle collisions, and, to less extent, cavitation. Cavitation is known to induce formation and collapse of microbubbles around graphite flakes, producing microjets and shock waves that propagate through nanographite volumes and contribute to better exfoliation.^12^

The interparticle collisions and the impact of particles on the channel walls are highly energetic processes, resulting in heat generation. To maintain room temperature during the process, the suspension circulates through a serpentine cooling system.^12^ Both the interaction chamber and the cooling serpentine were immersed in a water bath. We defined as one cycle the complete passage of the precursor suspension through the interaction chamber. To initiate subsequent cycles, the collected suspension was reintroduced into the inlet. In this work, we studied the exfoliation of graphite flakes after one, six, and ten cycles. For simplicity, we refer to exfoliated graphite as flakes that underwent the whole methodology proposed, that is, probe ultrasonication followed by microfluidization.

### Exfoliated Graphite Inorganic Composite

Once the graphite was exfoliated in suspension, sodium silicate (Na_2_SiO_3_) was incorporated to form the inorganic-based composite. Sodium silicate, also known as liquid vitreous, is water-soluble and transparent, making it attractive for photonic applications.^17,20^ The mixture was homogenized for 20 min using a mixer (RW2 digital, from IKA, Germany) at 200 rpm.

The concentration of sodium silicate in the composite is a critical parameter, as excessive amounts can induce the formation of exfoliated graphite clusters and also crystallization in the form of round-shaped clusters and needles, as shown in Figure 3. To minimize this effect, we used a Na_2_SiO_3_ concentration as low as 0.25%.

**Figure 3:**
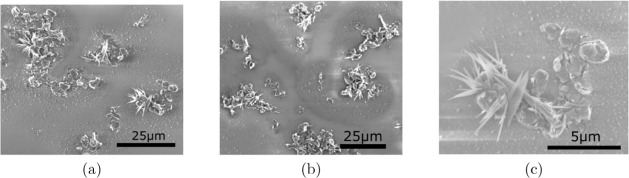
Examples of the crystallization effect in a graphite film with 1% sodium silicate concentration and annealed at 50 °C for 20 min.

During the exfoliation, the flakes might suffer aggressive breakage, generating undesired smaller nanographite fragments called debris, as shown in Figure 4. After the composite was prepared, centrifugation was employed to reduce the amount of debris in the composite. The centrifugation process also controls the graphite concentration in the composite, which is an advantage since many practical applications^12^ require higher material densities. The initial mass concentration of 0.05% was chosen to avoid channel occlusions during microfluidization.

**Figure 4:**
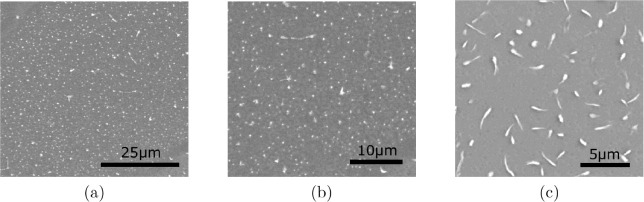
Examples of depositions made with supernatant debris (the upper part of the suspension) after centrifugation of the composite with 0.05% exfoliated graphite and 0.25% sodium silicate. The films of debris were prepared by drop casting and annealed at 30 °C for 20 min.

We centrifuged the composite for 3 min at 8000 rpm using the Eppendorf Centrifuge MiniSpin (Eppendorf, Germany). With this process, the exfoliated graphite was concentrated at the bottom and the supernatant debris remained in the upper part of the suspension. Hence, we can control the concentration of graphite by removing the upper part of the suspension, including the debris. To obtain a nanographite concentration of 0.1%, approximately 80% of the upper liquid phase should be removed.

## Results and Discussion

To investigate the composite properties, thin films were fabricated on glass substrates (20 mm × 20 mm, Exacta) by drop casting. After deposition, the films were baked at 30 °C for 20 min to remove residual solvents. We evaluated their surface interaction, composition, and morphology using Raman spectroscopy, scanning electron microscopy (SEM) and atomic force microscopy (AFM) analysis. In addition, the electrical conductivity of the films was measured by using a four-probe setup.

### Surface Interaction of Exfoliated Graphite Films

To evaluate the surface interaction of the exfoliated graphite via microfluidization, we used drop-casted films with different graphite concentrations in the composite, ranging from 0.05% to 0.3%. During the film formation, we verified that the contact angles were below 45°  for all concentrations analyzed, indicating a strong interaction with the surface, good adhesiveness, and wettability.

The exfoliated graphite films are shown in Figure 5a and b. At concentrations of 0.05 % and 0.2 %, the diameters obtained were 0.8 cm and 0.9 cm, respectively. For comparison purposes, we also prepared films using nonexfoliated graphite composites and there were no significant changes in the diameters, as shown in Figure 5c and d. For concentrations of 0.05 % and 0.2 % of nonexfoliated graphite films, the measured diameters were 0.7 cm and 0.8 cm, respectively.

**Figure 5:**
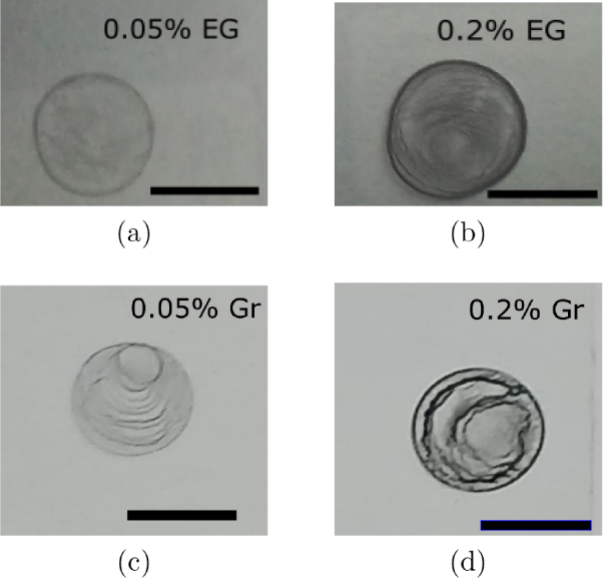
Films obtained with nanographite in two forms: (a, b) exfoliated graphite and (c, d) nonexfoliated graphite. The concentration of graphite was 0.05% for (a, c) and 0.2% for (b, d). The labels “EG” and “Gr” stand for exfoliated and original graphite, respectively. The scale bars are 1 cm.

The consistency of the diameters from the exfoliated graphite and nonexfoliated graphite films indicates that the formulated inorganic-based composite on a sodium silicate matrix with DI water and surfactant provides satisfactory surface interaction. Moreover, the mixture can form graphite films with excellent adhesiveness, as foreseen in^17^.

The improved dispersion of exfoliated flakes is evident in Figure 5. When comparing films of exfoliated and nonexfoliated graphite, the coffee-ring effect is more pronounced in nonexfoliated graphite films in both concentrations, whereas this effect is less dominant in exfoliated graphite films, resulting in higher uniformity.

### Compositional Analysis

We evaluated the composition of the film using Raman spectroscopy to identify the fingerprints of the two main components: exfoliated graphite and sodium silicate.

Using Raman XploRA (Horiba, USA) at 532 nm with a diffraction grating of 1800 grids/mm, we initially verified the spectrum of a pure sodium silicate sample. In the literature,^21^ it is known that the peaks of sodium silicate shift depending on the Na_2_O contents and the temperature of preparation. The peaks that suffer the most significant shifts with these parameters are above 800 cm^–1^. However, there are characteristic lower-frequency bands attributed to the symmetric bending of the silicate network that are less affected by Na_2_O contents and temperature. Hence, we focused on identifying the lower-frequency bands of the sodium silicate used in this work. The results that we obtained are shown in Figure 6. The most prominent peak is at 420 cm^–1^. When analyzing the film formed by the exfoliated graphite composite with sodium silicate, we observed that the peak at 420 cm^–1^ remained. Two other peaks at 1348 cm^–1^ and 1574 cm^–1^ appeared on the spectrum of the film, and they correspond to the D and G bands of graphite,^17^ respectively. The aspect ratio between the bands (*I*_*D*_/*I*_*G*_) indicates the quality of the graphite.^14^ We obtained a ratio of 0.22, which indicates a low level of defects between the graphene layers and agrees with values earlier reported in the literature.^15,22^

**Figure 6:**
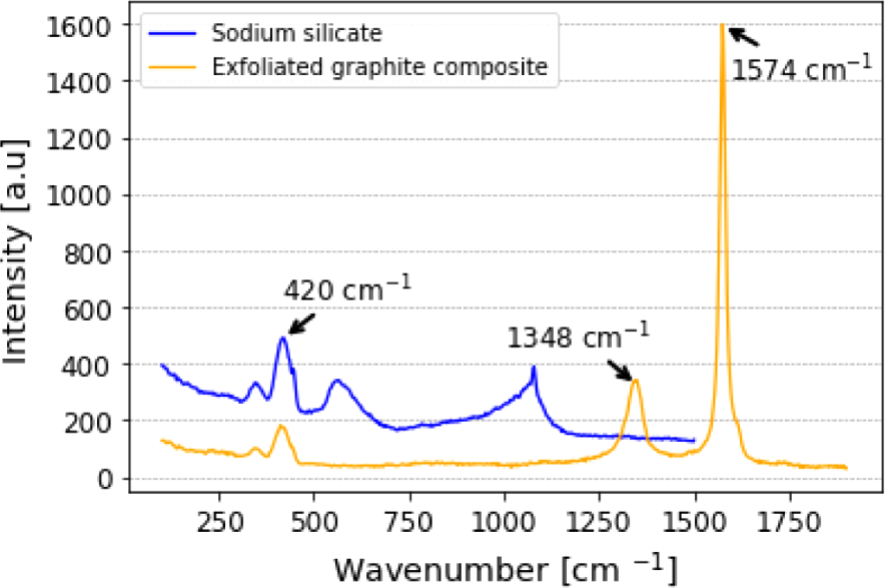
Raman spectra of sodium silicate and the exfoliated graphite composite. The blue line shows a characteristic peak of sodium silicate at 420 cm^–1^. In the orange line, we observe the D and G bands of the graphite at 1348 cm^–1^ and 1574 cm^–1^, respectively, along with the peak of sodium silicate.

Even though the Raman spectrum of sodium silicate depends on its processing, we can clearly see its fingerprint in the spectrum of the film formed by the graphite composite. Moreover, this analysis highlighted that the microfluidization process maintained the high quality of the graphite used.

### Morphology of Exfoliated Graphite Films

Beyond the macroscopic aspects discussed in the previous sections, the morphology of the films was analyzed with SEM to compare the distribution of the flakes deposited on the substrate with different graphite concentrations in the composite.

We investigated films with 0.05% and 0.1% of exfoliated graphite with sodium silicate, as shown in Figure 7a and b. For the lower concentration, the density of nanographite flakes was 0.014 flakes/μm^2^. When increasing the concentration 2-fold, the density increased accordingly to 0.031 flakes/μm^2^, confirming a consistent scaling content. These results confirm that the centrifugation process described above is an effective strategy to control the density of exfoliated graphite flakes in the composite.

**Figure 7:**
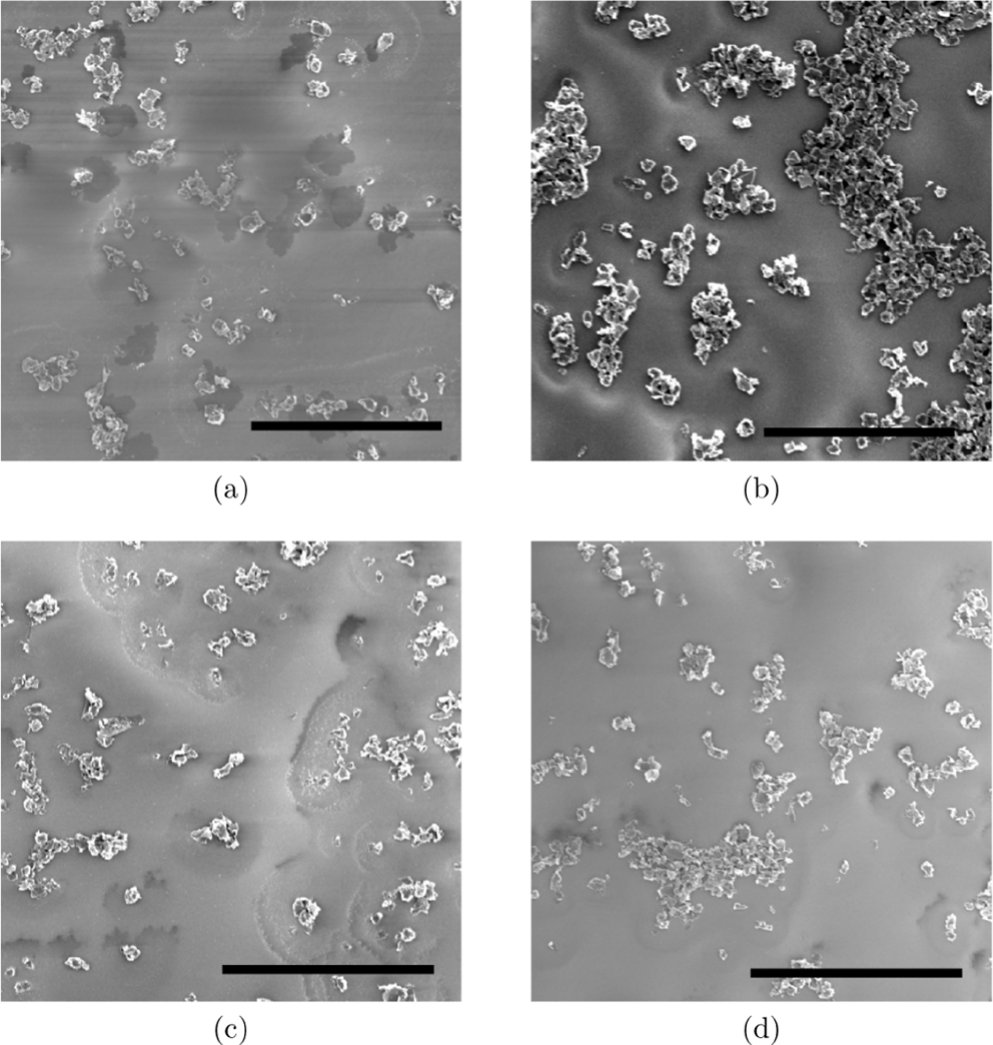
Exfoliated nanographite films were formed by drop casting from the proposed composite. In (a) and (b), we utilized one microfluidization cycle for graphite concentrations of 0.05% and 0.1%, respectively. In (c) and (d), we maintained the graphite concentration at 0.05% and used six and ten cycles for exfoliation. The scale bars are 50 μm.

For subsequent analyses, we maintained a low graphite concentration of 0.05% to facilitate the identification of individual flakes. At this concentration, we compared the flake densities obtained after different numbers of microfluidization cycles: 1, 6, and 10.

For the quantitative analysis, the density values were averaged over six distinct regions of each film, corresponding to a total analyzed area of 0.18 mm^2^. Representative SEM images of exfoliated nanographite after 6 and 10 cycles are shown in Figure 7c and d, respectively. The results are summarized in Table 2.

**Table 2:** Density of Exfoliated Graphite Flakes Deposited on a Substrate

Number of cycles	Density (number of flakes/μm^2^)	Graphite covered area (%)
Nonexfoliated	0.007	7.8
1	0.014	14.5
6	0.015	16.2
10	0.016	17.6

Exfoliation by microfluidization increased the flake density nearly 2-fold compared to the nonexfoliated graphite, from 0.007 flakes/μm^2^ to 0.014 flakes/μm^2^. Increasing the number of cycles further enhanced the density, although the effect was more modest, reaching up to 0.016 flakes/μm^2^ after ten cycles.

### Thickness and Sheet Size of Exfoliated Graphite

Considering the exfoliated graphite obtained after 10 cycles of microfluidization, the thicknesses of individual flakes were determined via AFM. In Figure 8, we show some typical AFM images of the thinner flakes and their respective line profiles. The aspect ratio (lateral dimension/height) ranged from 100 to 500, corresponding, respectively, to thicknesses of 10 nm to 2 nm, with lateral dimensions being around 1 μm. Each profile was fitted, and the average thickness obtained was 7 nm.

**Figure 8:**
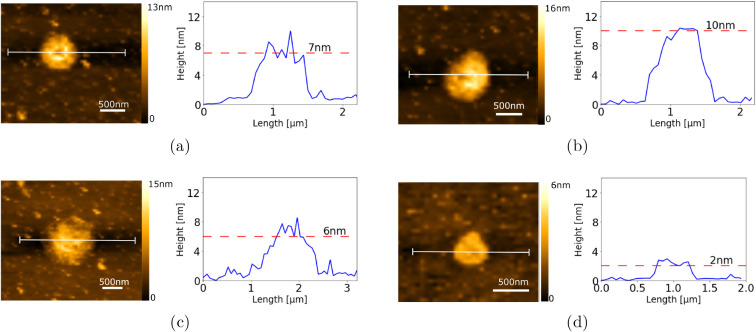
Typical AFM images (a–d) of exfoliated graphite flakes with 10 cycles of microfluidization. The color ruler represents the topography of the flakes. On the right of each image, we show the line profile and height of the respective flake.

Considering larger areas of exfoliated graphite films, we evaluated the lateral dimensions of 50 exfoliated nanographite flakes shown in the SEM images in Figure 7. We observed that exfoliation by microfluidization tends to preserve the lateral sheet size of the flakes from the precursor suspension. As shown in Figure 9 and summarized in Table 3, Gaussian-like fits of the frequency distribution of the lateral size of the flakes before and after the microfluidization and centrifugation processes revealed average sheet sizes of 2.73 μm, 2.74 μm, and 2.40 μm for 1, 6, and 10 cycles, respectively. These values are consistent with the unprocessed sheet size of 2.83 μm, confirming that microfluidization primarily reduces the flake thickness without significant impairments to the sheet size. As previously discussed, probe ultrasonication varies the fwhm compared to the original distribution. Following microfluidization, however, the fwhm values converged toward those of the original distribution, indicating a recovery of the flake size uniformity.

**Figure 9:**
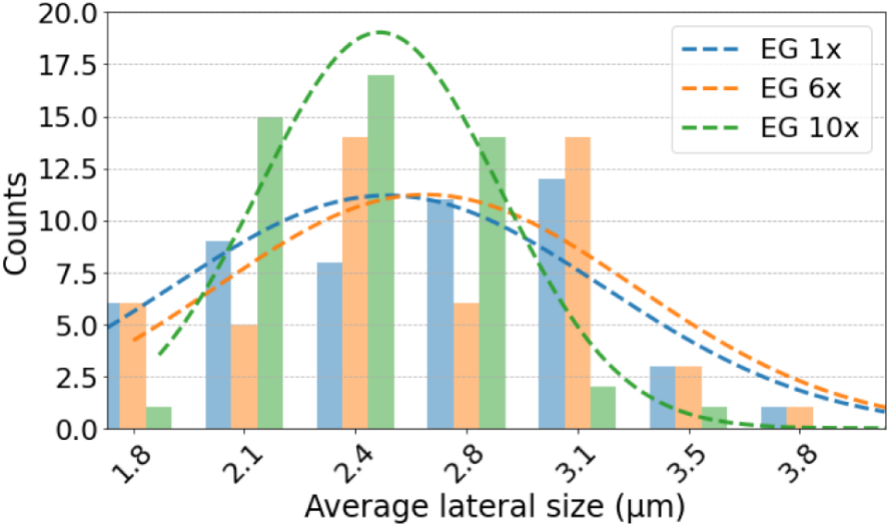
Gaussian-like distribution of exfoliated nanographite sheet size. The label EG stands for exfoliated graphite. The labels also indicate the number of microfluidization cycles (1, 6, and 10).

**Table 3:** Influence of Microfluidization Cycles on the Exfoliation of Graphite Flakes

Process	Size (μm)	fwhm (μm)
Non processed	2.83	1.16
Exfoliated with 1 cycle	2.73	1.28
Exfoliated with 6 cycles	2.74	1.28
Exfoliated with 10 cycles	2.40	0.85

Thus, we conclude that microfluidization conserves the flake size and enhances the aspect ratio. In combination with centrifugation, the amount of debris and damaged flakes can be reduced, resulting in better-quality nanographite at the end of the process.

### Electrical Properties of Exfoliated Graphite

One of the interesting properties of graphene is its high electrical conductivity. However, achieving such values on the macroscopic scale is still an open challenge.^23^ In a graphene film, the spaces between the flakes can seriously affect the conductivity. For this reason, thicker films of exfoliated nanographite were required for conductivity experiments. Using only exfoliated graphite obtained after 5 cycles of microfluidization, we prepared films via vacuum filtration.^19^ With this filtration technique, we obtained thicker films with denser regions composed of multiple layers of exfoliated graphite.

The suspension containing exfoliated graphite in water and surfactant was filtered on a proper paper (0.22 μm pore size) substrate with a diameter of 20 mm (GSWP type, Millipore, Germany). During filtration, suspension was repeatedly rinsed with water to remove the remaining surfactant, as it could affect conductivity. Then, the film deposited on the paper was dried at room temperature for 24 h and subsequently pressed with a uniaxial press at a load of 3 tons for 30 min to achieve a leveled surface. The final thickness of the film was 17 μm, with a density of nearly 1.5 g/cm^3^. We measured the resistance of the so obtained films at three different regions with the four-probe technique using a high precision electrical source meter (B2912A, Agilent, USA). The resistivity of each region was calculated following ref 24, and the averaged value was estimated to be as low as 0.02 Ω cm, indicating the high quality of the produced nanographite flakes.

## Conclusion

In this work, we detail an exfoliation methodology based on microfluidization to achieve nanographite flakes with only a few dozen layers. Motivated by previous studies,^17^ we propose the incorporation of the exfoliated graphite into an inorganic composite with sodium silicate. This composite is compatible with several thin-film deposition techniques, thereby broadening the range of functionalities and applications of exfoliated nanographites.

Our results clearly show that probe ultrasonication with longer duration, while being effective in exfoliation processes, also introduces undesirable effects such as flake fragmentation, surface damage, and debris generation. The integration of microfluidization alleviates these issues. Using a prior and shorter sonication time of only 5 min, it is possible to initiate the exfoliation to prevent microfluidizer channel occlusions. The subsequent microfluidization achieved thinner flakes: from 2 nm to 10 nm, maintaining micrometric lateral dimensions. Hence, the proposed methodology resulted in graphite flakes with aspect ratios of up to 500 and reduced the thickness of the original graphite powder by an average of 40 times.

Furthermore, we anticipate applying the exfoliated graphite-based composite for film deposition via atomization-based techniques.^25^ Moreover, the reduced opacity observed in the films and the controlled concentration of graphite in the composite suggest strong potential for optics and photonics applications, where transparency and uniformity are critical.
